# Predictors of multiglandular disease in primary hyperparathyroidism

**DOI:** 10.1007/s00423-017-1647-9

**Published:** 2018-01-02

**Authors:** Mark Thier, Sébastien Daudi, Anders Bergenfelz, Martin Almquist

**Affiliations:** 1 Department of Clinical Sciences, Lund University, Lund, Sweden; 2 Department of Surgery, Skane University Hospital Lund University, SE-221 85 Lund, Sweden

**Keywords:** Primary hyperparathyroidism, Multiglandular disease, Negative scintigraphy, Prediction, Parathyroid hyperplasia

## Abstract

**Background:**

Primary hyperparathyroidism (pHPT) is caused by single- or multiglandular disease (MGD). Patients with MGD have an increased risk of complications at surgery and for persistence and recurrence after surgery. The study evaluated whether preoperative clinical and biochemical characteristics could predict MGD in patients with pHPT.

**Methods:**

We retrospectively evaluated patients operated 1989–2013 for first-time, non-hereditary pHPT. MGD was defined in patients with more than one pathological gland excised at surgery or with persistent hypercalcemia after the excision of a single pathological parathyroid gland, confirmed by histopathology. Clinical and biochemical variables were compared in patients with single- and multiglandular disease. Logistic regression was used to identify variables predicting MGD, yielding odds ratios (OR) with 95% confidence intervals (CI).

**Results:**

There were 707 patients, of which 79 (11%) had MGD. Patients with MGD were more likely to have negative sestamibi scintigraphy than patients with single-gland disease, 15 of 49 (31%) vs. 70 of 402 (17%; *p* = 0.03), to suffer from diabetes (12 of 74, 16%) vs. 45 out of 626 patients (7.2%; *p* < 0.01) and had lower preoperative levels of urinary calcium (3.80 vs. 4.44 mmol/L; *p* = 0.04). Multivariable analysis identified negative scintigraphy (OR 2.42; 95% CI 1.18 to 4.79), diabetes (OR 2.75; 95% CI 1.31 to 4.97) and elevated levels of osteocalcin (OR 3.79, 95% CI: 1.75 to 8.21) as predictors of MGD.

**Conclusion:**

Negative sestamibi scintigraphy, diabetes and elevated osteocalcin levels were predictors of MGD.

## Introduction

Sporadic primary hyperparathyroidism (pHPT) is caused by a single-gland adenoma in the majority of patients. However, the incidence of multiglandular disease (MGD) has been shown to vary in the range 7–23% [1].

PHPT caused by single-gland disease (SGD) is cured by simple resection of the parathyroid adenoma, whereas pHPT caused by MGD requires bilateral neck exploration with increased risks, including postoperative hypocalcemia, neck hematoma and recurrent laryngeal nerve palsy [2]. Additionally, operating times, postoperative pain, average hospital stay and costs are increased following bilateral neck exploration [2]. Furthermore, patients with MGD have a lower likelihood of cure after surgery than patients with SGD [3].

To counsel patients about the risks and benefits of surgery for pHPT, and to plan for the least invasive surgery, it would be of great benefit to predict MGD preoperatively.

Neck ultrasonography and sestamibi scintigraphy are the most common modalities to distinguish between SGD and MGD, with reported positive predictive values of 80–90% [4, 5]. Concordant ultrasonographic and scintigraphic results are highly predictive for solitary adenomas, almost approaching 100% [4], whereas negative localisation studies are associated with a higher risk for MGD [6]. Another study found that neurocognitive symptoms could predict MGD in patients with pHPT [9].

Several studies have tried to develop scores to predict MGD [6–9]. The Wisconsin index [7] yields the intraoperative probability of remaining hyperfunctioning gland(s) after the resection of one gland. This index consists of preoperative serum calcium and PTH levels and intraoperative parathyroid gland weight, and inherently, it cannot be used to predict MGD preoperatively.The CaPTHUS score combines the results of ultrasound, scintigraphy, serum calcium, levels of parathyroid hormone (PTH) and urinary calcium [10]. The score was found to accurately predict SGD by one group [11], whereas several other groups found it reliable only in patients with overt disease and positive imaging studies [7, 12]. Contemporary patients with pHPT are more likely to be diagnosed with minor or no symptoms and mild disease, hence the CaPTHUS score might be less reliable in a majority of the patients. Mild disease and low adenoma weight seem more likely to occur with multiple gland disease [1, 10, 37], thus further emphasising the difficulty in preoperative prediction of MGD.

Due to the lack of reliable, strictly preoperative predictors for MGD, we aimed for identification of such factors within our patient cohort.

It has been speculated that SGD and MGD are different disease entities, but little is known about the etiology of MGD.

This study was designed to identify clinical, biochemical and radiological markers to predict MGD in patients with pHPT.

## Methods

Data on consecutive patients operated upon for pHPT at Lund University Hospital have been collected in a database since 1989. The database contains information about the patients’medical history, clinical variables and details on surgery, histology and follow-up. Patients with diabetes had a medical history of diabetes, independent of their treatment.

For the present study, patients with recurrent pHPT, secondary hyperparathyroidism, ongoing or prior lithium medication, multiple endocrine neoplasia (MEN) syndrome, familial hyperparathyroidism, familial hypocalciuric hypercalcemia, parathyroid carcinoma and a follow-up time of < 12 months were excluded.

### Outcome

Cure was defined as calcium levels below the upper level for normocalcemia (ionised calcium < 1.35 mmol/L (< 5.40 mg/dL) or total serum calcium < 2.50 mmol/L (10.0 mg/dL) 12 months after surgery, regardless of PTH values. Routine follow-up is conducted at 4 weeks and 12 months after surgery.

Persistent disease was defined as hypercalcemia within 12 months from date of surgery. Recurrent disease was defined as hypercalcemia that occurred more than 12 months after surgery.

MGD was defined as (a) more than one enlarged parathyroid gland excised at surgery and confirmed by histopathology regardless of cure or (b) when the patient remained hypercalcemic after the excision of a single enlarged parathyroid gland, confirmed by histopathology.

SGD was defined as cured after the excision of one pathological gland.

### Preoperative localisation studies

All patients with pHPT, referred to our unit are routinely referred to ultrasound of the neck and sestamibi scintigraphy. The results of the scintigraphic studies were encoded as nominal data; positive for SGD, negative or missing.

Sestamibi scintigraphy was introduced for routine use in our department between 1994 and 1995. Due to the lack of radiologic expertise, the access to neck ultrasound was significantly limited during the study period.

### Biochemistry

The following biochemical markers were analysed preoperatively: calcium, ionised calcium, PTH, phosphate, ALP, osteocalcin, urinary calcium (U-Ca), 25(OH)D_3_, fasting blood glucose and iohexol clearance.

Serum ionised calcium concentrations (reference range 1.15–1.35 mmol/L; 4.60–5.40 mg/dL) were analysed from blood samples normalized to pH 7.4 with the ion-selective electrode ABL 505 (Radiometer, Copenhagen, Denmark). The method has a coefficient of variation (CV) of < 1% at an assigned value of 1.27 mmol/L. Levels of total serum calcium (reference range 2.20–2.50 mmol/L; 8.80–10.0 mg/dL) were measured by a routine laboratory analyzer.

Alkaline phosphatase (ALP) was measured bichromatically at 450 and 480 nm at an alkaline pH, reference range 0.60–1.8 μmol/L (35.9–107.8 U/L). The CV for this method is 6.9% at 0.57 μmol/L.

Phosphate was measured bichromatically at 340 and 700 nm. The concentration was determined by the difference in absorbance. The reference range was 0.8–1.5 mmol/L (2.48–4.64 mg/dL). The method has a CV of 5.8% at 0.7 mmol/L.

Osteocalcin was measured through one-step immunometric sandwich assay using ElectroChemiLuminiscence Immunoassay based on a derivate of Reuthenium (Ru). The reference range was 10–43 μg/L (adults). The CV for this method is 3% at 19 μg/L.

Urinary calcium (U-Ca) was measured after the formation of calcium complexes in two steps, with 5-nitro-5′-metyl-BAPTA (NM-BAPTA) followed by EDTA. The sum of ionised calcium and calcium complexes was measured bichromatically at 376 and 340 nm. The method has a CV of 1.2% at 1.8 mmol/L and 1.0% at 2.6 mmol/L. No reference range exists for U-Ca, but for tU-Ca the range is 2.5–7.5 mmol/24 h (10.0–30 mg/dL).

Plasma parathyroid hormone, PTH, was analysed by an assay for intact PTH (Hitachi Modular –E), with a reference range of 1.6–6.9 pmol/L (15.1–65.1 ng/L). The analysis had a total CV of 5.9% at 100 pmol/L. On 20 March 2000, the method was changed to an assay for intact PTH (Hitachi Modular –E), with a reference range 1.6–6.9 pmol/L. Due to the change of methods, a correction algorithm was used between the old and new values: new value = 1.4 × old value = 0.2, as defined by the Department of Clinical Chemistry at Lund University Hospital.

High-performance liquid chromatography was used for assessment of the level of serum 25-hydroxyvitamin D_3_, (25(OH)D_3_). The CV for 25(OH)D_3_ is 15% at 50 nmol/L.

The glomerular filtration rate (GFR), was determined by a technique that measures renal clearance of the contrast agent iohexol. The average value for young healthy subjects is 127 mL/min with a reduction in subjects older than 55. In 65-year-old subjects, the expected GFR is approximately 80 mL/min.

### Bone mineral density

Since 1994, bone mineral density (BMD) of the lumbar spine (L2–L4), the femoral neck and shaft, and distal third of the radius has been investigated by dual-energy X-ray absorptiometry (DXA). In this study, measurements were made with Lunar Expert XL equipment, software version 1.72 (Lunar Corp, Madison, Wis). The method has a CV of 1%. Bone mineral density is expressed in grams per square centimeter (g/cm^2^) and as age- and gender-specific standard deviations (*Z*-scores).

Bone densitometry was carried out preoperatively giving a BMD *Z*-score for the lumbar spine, femoral neck and distal 1/3 of the radius.

### Missing data

Continuous variables with less than 10 % missing values, except for values of ionised calcium, had missing values replaced with the median [13]. This was done for phosphate, ALP and PTH. For missing values of ionised calcium, a conversion factor (ionised ca/total calcium) was calculated, using values of total calcium, which had no missing data. Osteocalcin, U-Ca, 25(OH)D_3_, iohexol clearance and BMD *Z*-score each had more than 10 % missing values. These variables were categorized into tertiles, with ‘missing’ as a separate category.

Neck ultrasonography was performed on less than half of the patients due to the lack of radiological expertise and therefore, we opted not to include information on ultrasonography in the study.

### Statistical analysis

All variables were checked for normality, using histograms and box plots. The cohort was analysed and stratified as SGD or MGD. Medians with interquartile range (IQR) were calculated for continuous data in the two subgroups. Continuous data were compared using the two-sample Student’s *t* test and the Mann-Whitney *U* test for normal and skewed distributions where appropriate. Pearson’s chi-square test was used to compare categorical data between groups. Univariable and multivariable logistic regression models were developed to identify preoperative factors independently associated with MGD. The following independent variables were included in the analysis: gender, positive scintigraphy (yes or no), diabetes (yes or no), age, ionised calcium, phosphate, ALP, PTH, U-Ca, osteocalcin, iohexolclearance, 25(OH)D_3_, and BMD *Z*-score for the radius. Odds ratios (OR) and 95% confidence intervals (CI) were calculated. A *p* value < 0.05 was considered statistically significant. All tests were two-tailed. STATA Special Edition version 13.1 was used.

## Results

A total of 837 individuals underwent surgery for pHPT at Lund University Hospital during the time period 1989–2013. Out of these, 707 patients were included in the present study, 546 women (77%) and 161 men (23%). Some 628 patients had SGD (89%) and 79 patients had MGD (11%).

Characteristics for patients with SGD and MGD are shown in Table 1. Gender, age, BMD *Z*-score or biochemical presentation did not differ between patients with MGD and SGD.

**Table 1:** Characteristics of patients with single- and multiglandular disease. Categorical data presented as number and column per cent, *n* (%). Continuous data presented as medians and interquartile range (IQR). Significant results are marked in bold

	Single gland (*n* = 628)	Multigland (*n* = 79)	
Characteristics	*n* (%), median (IQR)	*n* (%), median (IQR)	*p*
Gender			
Female	490 (78.0)	56 (70.9)	0.15
Male	138 (22.0)	23 (29.1)	
Sestamibi scintigraphy			
Positive	332 (52.9)	34 (43.0)	**0.03**
Negative	70 (11.2)	15 (19.0)	
Not performed	226 (35.9)	30 (38.0)	
Diabetes			
Yes	45 (7.2)	12 (16.2)	**< 0.01**
No	581 (92.8)	62 (83.8)	
Age (years)	*65 (56–74)*	*68 (56–76)*	0.21
Ionised calcium (mmol/L)	1.45 (1.40–1.52)	1.46 (1.38–1.52)	0.83
Phosphate (mmol/L)	0.79 (0.70–0.90)	0.79 (0.66–0.88)	0.48
Alkaline phosphatase (μkat/L)	1.80 (1.30–3.0)	1.70 (1.20–2.40)	0.17
PTH (pmol/L)	9.90 (7.30–13.0)	10.0 (8.50–15.0)	0.09
U-Ca (mmol/L) (26%)*	4.44 (2.80–6.60)	3.80 (2.80–5.10)	**0.04**
Osteocalcin (μg/L) (18%)*	30.0 (18.0–46.0)	33.0 (26.0–49.0)	0.06
Iohexol clearance (mL/min), (26%)*; SI	78.0 (65.0–90.5)	72.50 (60.0–93.0)	0.31
25(OH)D (nmol/L) (17%)*	50.0 (37.0–65.0)	46.0 (35.0–57.0)	0.23
BMD *Z* radius (g/cm^2^) (36%)*	− 0.60 (1.60–0.30)	− 0.40 (− 1.50–0.70)	0.39

*Missing % if > 1%

Of 85 patients with negative scintigraphy, 15 (18%) had MGD, compared to 34 of 366 patients (9%) with positive scintigraphy (*p* = 0.03). Patients with MGD were more likely to suffer from diabetes than patients with SGD (12 of 79; 15%) vs. (45 of 628; 7%; *p* < 0.01) and had lower preoperative levels of urinary calcium; median 3.80 mml/L vs. 4.44 mol/L; *p* = 0.04 (0.095 vs. 0.11 mg/dL). Glucose levels did not differ between the groups.

Among the 15 patients with negative sestamibi scintigraphy and MGD, one suffered from diabetes. Conversely, among 366 patients with positive scintigraphy, 332 had single-gland disease. Among those, 311 (94%) did not suffer from diabetes.

### Logistic regression

Table 2 shows the results of the logistic regression analyses. In univariable analysis, MGD was associated with a negative sestamibi scintigraphy (OR 2.09, 95% CI: 1.08 to 4.05) as well as with osteocalcin, 2nd tertile (OR 3.12, 1.51 to 6.45) and 3rd tertile (OR 1.95, 0.90 to 4.22).

**Table 2:** Results of univariable and multivariable logistic regression analysis. Odds ratio and 95% confidence interval are presented. Odds ratios are calculated for the outcome multiple gland disease. For categorical variables, the reference category has an odds ratio of 1.00. The table shows calculated values for 707 patients that comprised the study population. Significant results are marked in bold

	Univariate logistic regression		Multivariate logistic regression	
Characteristics	OR	95% CI	OR	95% CI
Gender				
female	1.00			
male	1.46	0.87–2.46	1.42	0.70–2.85
Sestamibi Scintigraphy				
positive	1.00		1.00	–
negative	**2.09**	1.08–4.05	**2.32**	1.13–4.78
Diabetes				
Yes	**2.50**	1.26–4.97	**2.97**	1.40–6.30
No	1.00		1.00	
Age	1.01	0.99–1.03	1.00	0.98–1.02
Ionised calcium	0.78	0.08–7.15	0.32	0.28–3.65
Phosphate	0.58	013–2.59	0.71	0.12–4.24
Alkaline phosphatase	0.88	0.73–1.04	0.76	0.43–1.35
PTH	1.01	0.99–1.02	1.39	0.82–2.38
U-Ca				
1st tertile	1.00			
2nd tertile	1.30	0.69–2.44	1.26	0.63–2.49
3rd tertile	0.57	0.27–1.21	0.34	0.10–1.14
Osteocalcin				
1st tertile	1.00			
2nd tertile	**3.12**	1.51–6.45	**3.39**	1.75–8.21
3rd tertile	1.95	0.90–4.22	2.11	1.02–5.48
Iohexol clearance				
1st tertile	1.00			
2nd tertile	0.67	0.35–1.27	0.65	0.32–1.35
3rd tertile	0.68	0.36–1.29	0.57	0.21–1.02
25(OH)D_3_				
1st tertile	1.00			
2nd tertile	0.93	0.51–1.68	0.86	0.45–1.65
3rd tertile	0.58	0.29–1.14	0.49	0.22–1.04
BMD *Z* radius				
1st tertile	1.00			
2nd tertile	1.13	0.55–2.32	1.04	0.48–2.24
3rd tertile	1.34	0.66–2.6	1.21	0.56–2.59

*OR*, odds ratio; *CI*, confidence interval; *PTH* parathyroid hormone; *BMD* bone mineral density; *25(O)D3*, 25 hydroxy vitamin D3; *U-Ca* urinary calcium

In multivariable, logistic regression, diabetes (OR 2.97, 95% CI 1.40–6.30), osteocalcin (2nd tertile OR 3.39 CI 1.75–8.21, 3rd tertile OR 2.11, 95% CI 1.02–5.48) and negative scintigraphy (OR 2.32, 95% CI 1.13–4.78) were all independently associated with MGD.

## Discussion

In this study on 707 patients with sporadic, first-time primary hyperparathyroidism, diabetes was significantly associated with multiglandular disease (MGD), both in univariable and multivariable analysis. We are unaware of any previous report on such an association, but an association between pHPT overall and diabetes type 2 has been described [14, 15]. Also, physical inactivity was recently described as a risk factor for the development of pHPT [16]. The present study cannot answer whether MGD contributes to the development of diabetes, or if diabetes causes MGD. However, the parathyroid glands are closely linked to glucose metabolism. Parathyroid hormone, insulin and osteocalcin interact by modulating insulin secretion, sensitivity and peripheral lipolysis [17–19]. Osteocalcin was identified as an independent risk factor for MGD, which raises questions about whether MGD and SGD differ in their impact on bone mineral density or if this correlation rather represents differences in metabolic changes between the two entities. The second tertile had a stronger correlation than the third which could point towards a multifactorial and complex biological role of osteocalcin in the development of MGD rather than a simple cause and effect correlation. Further research in the field of bone and glucose metabolism and its impact of the pathophysiology of the parathyroid glands is needed.

Recent research [20–22] suggests that growth factors, for instance insulin-like growth factor-1 (IGF-1), FGF-23 and vascular endothelial growth factor (VEGF) not only modulate insulin sensitivity but also play a role in the development of parathyroid adenomas and hyperplasia. It has been shown that IGF-1 is decreased in both parathyroid adenoma and hyperplasia, but no difference could be found between the immunoreactivity of the two entities [21].

Previous studies [23, 24] have suggested that parathyroid adenoma is a predominantly monoclonal lesion, while hyperplasia has been described as an oligo- or polyclonal lesion. It is possible to speculate that the parathyroid glands, due to insulin resistance, the action of insulin-like growth factors or by direct action of glucose levels, are stimulated to grow in a multiglandular fashion.

We also observed MGD in 18% of patients with negative sestamibi scintigraphy compared to 9% in patients with positive scintigraphy, which is in line with previous reports [25–32].

We found no difference in the preoperative ionised calcium and intact PTH levels between patients with SGD and MGD. Some groups have reported higher serum calcium and intact PTH levels in patients with SGD compared to MGD [6, 7, 10], while others could not confirm these findings [33]. In the present study, urinary calcium was significantly lower in patients with MGD, but was not associated with MGD in multivariable logistic regression. Schneider et al. reported a 35% incidence of MGD in patients with mild pHPT and low urinary calcium, more than double the rate in patients with overt disease [10]. The patients in our study were defined as having overt disease according to Schneider’s criterion, and probably therefore more likely to suffer from SGD.

There is a trend toward earlier surgical intervention in patients with pHPT, leading to fewer symptomatic patients even among patients with single-gland disease [34–40]. It remains unclear whether patients with mild disease are diagnosed earlier in the course of the disease, or if at least some of them belong to a group with a different etiology, caused by MGD.

No single factor, alone or in combination, could reliably predict MGD in patients with pHPT, in the present study, due to overlap. According to the results of the present study, patients with positive scintigraphy and without diabetes, are, however, highly unlikely to be diagnosed with MGD.

### Limitations

In the analysis of data, missing data were replaced by medians or treated as a separate category. This might have obscured associations between predictive factors and MGD. Missing data that occur due to a systematic dropout or non-responsiveness of a certain patient category could obscure associations between predictive factors and MGD. We believe that our data are missing at random and therefore still representative for the whole population.

Information on the results of ultrasonography was not included in the analysis since it was performed in less than half of the patients. This being a single-institution study, albeit covering a large time period, the number of patients included in the study could have been too small to evaluate the difference between patients with single-gland disease and MGD.

## Conclusion

Diabetes and increased levels of osteocalcin were associated with MGD, although negative localisation studies remain the most clinically usable predictors.
